# Asparaginase Hypersensitivity Reactions in NK/T-Cell Lymphomas

**DOI:** 10.3390/clinpract15110211

**Published:** 2025-11-17

**Authors:** Javier Varela Gonzalez-Aller, Pablo Nadal, Salome Cañizares, Carmen Muñoz, Anna Valer, Eva Gonzalez-Barca, Eva Domingo, Ana Sureda, Silvana Novelli

**Affiliations:** 1 Pharmacy Department, Catalan Institute of Oncology, L’Hospitalet de Llobregat, 08908 Barcelona, Spain; javiervarela@iconcologia.net (J.V.G.-A.);; 2 Hematology Department, Institut Català d’Oncologia, L’Hospitalet de Llobregat, 08908 Barcelona, Spain; 3 Bellvitge Biomedical Research Institute (IDIBELL), L’Hospitalet de Llobregat, 08908 Barcelona, Spain

**Keywords:** NK/T-cell lymphoma, asparaginase, hypersensitivity, drug allergy, PEG-asparaginase, crisantaspase, neutralizing antibodies

## Abstract

**Background/Objectives**: Asparaginase (ASP)-based chemotherapy has substantially improved clinical outcomes in Epstein–Barr virus (EBV)-positive NK/T-cell lymphomas (NKTCL). However, as a bacterial-derived enzyme, ASP is frequently associated with immune-mediated adverse events, particularly hypersensitivity reactions (HSRs), which may compromise both treatment efficacy and patient safety. This report presents a case of an ASP-related HSR and reviews the incidence within our institutional cohort. **Detailed Case Description**: A 60-year-old female presented an immediate Grade 2 HSR during her second PEG-asparaginase infusion, with pruritus, vomiting, and presyncope. The infusion was discontinued, and she was subsequently transitioned to crisantaspase—an alternative formulation—which was well tolerated without further adverse events. She remains disease-free to date. A retrospective review of institutional records (2015–2025) identified six patients with NKTCL treated with ASP-containing chemotherapy. The incidence of HSRs in this cohort was 1 of 6 (16.7%). **Conclusions**: As in acute lymphoblastic leukemia, HSRs to asparaginase remains a major challenge in the management of NKTCL with potential implications for treatment safety and efficacy. The establishment of standardized, consensus-based criteria for the diagnosis, classification, and management of ASP-related HSRs is urgently needed to optimize patient outcomes.

## 1. Introduction

Epstein–Barr virus (EBV)-positive NK/T-cell lymphomas (NKTCL) are highly aggressive neoplasms encompassing both EBV-positive nodal T- and NK-cell lymphoma and extranodal NK/T-cell lymphoma, formerly referred to as the nasal type. These malignancies represent approximately 10% of non-Hodgkin lymphomas (NHL) in Asia and several Latin American countries, but are rare in the United States, accounting for only 1–2% of NKTCL and just 0.2% of all NHL. Their incidence is, however, markedly higher among Hispanic and Asian/Pacific Islander individuals. A recent German study reports an annual incidence of 0.77 per 100,000 people [1,2,3].

Multiple prognostic scores have been described to stratify patient outcomes. Recently, the incorporation of β-2-microglobulin into the Prognostic Index of Natural Killer Lymphoma (PINK-B) has been shown to improve discrimination [4], as significant differences in three-year outcomes were observed among risk groups. Overall survival (OS) reached 84.1% in low-risk, 46.8% in intermediate-risk, and 17.6% in high-risk patients, While 3-year progression-free survival (PFS) showed a similar gradient, at 70.6%, 35.9%, and 7.3%, respectively. Initially, overexpression of P-glycoprotein (P-gp) was thought to be the main driver of multidrug resistance in extranodal NK/T-cell lymphoma (ENKTCL) [5]. However, the poor efficacy of P-gp-independent agents, such as methotrexate and cytarabine [6], indicates that additional resistance mechanisms are involved, as reflected by the suboptimal clinical outcomes observed with these drugs.

In 2005, selective sensitivity to L-Asparaginasa of ENKTCL cells was demonstrated. Since the incorporation of asparaginase (ASP) into combination regimens, response rates and long-term outcomes have improved substantially. ASP catalyzes the hydrolysis of asparagine, and its depletion in plasma inhibits protein and nucleotide synthesis, ultimately inducing apoptotic death of tumor cells.

Despite its clinical benefit, ASP administration is limited by immune-mediated toxicity. As a bacterial enzyme, ASP can induce hypersensitivity reactions (HSR) ranging from mild cutaneous manifestations to severe anaphylaxis. Most data on ASP-related hypersensitivity derive from acute lymphoblastic leukemia (ALL), where the reported incidence ranges between 10 and 30% for native E. coli derived ASP and 3–24% for pegylated formulations. However, data on NKTCL remain scarce, even though ASP-containing regimens such as SMILE, LVP, and P-GemOx are increasingly used as frontline or salvage therapies [7,8,9].

ASP is a bacterial-derived catalytic enzyme available in three main formulations: L-Asparaginase (L-ASP), PEGylated-Asparaginase (PEG-ASP), and crisantaspase (CSP). Both L-ASP and PEG-ASP are derived from Escherichia coli; however, pegylation significantly reduces the immunogenicity of PEG-ASP compared with native L-ASP, resulting in a lower incidence of hypersensitivity or anaphylactic reactions, particularly in patients without prior exposure to native formulations [10]. Crisantaspase, a less immunogenic Erwinia chrysanthemi-derived enzyme, is recommended in these cases to maintain efficacy while avoiding immune hyperreactivity [11].

Immune-mediated reactions and the development of neutralizing antibodies remain the primary limitations of ASP therapy. These antibodies may occur even in the absence of clinical symptoms, potentially compromising enzymatic activity and treatment efficacy. Nevertheless, evidence on the incidence, clinical features, and management of ASP-related HSRs in NKTCL remains scarce. Systematic evaluation and reporting of these reactions is needed to inform best practices, minimize treatment interruptions, and optimize patient outcomes.

Herein, we report the clinical presentation of an ASP-induced HSR in a patient with NKTCL and present a retrospective institutional review to determine its frequency.

## 2. Detailed Case Description

A 60-year-old previously healthy female was diagnosed with an ENKTCL-stage EIIA involving the left nasal cavity. She was scheduled to receive “sandwich” therapy consisting of chemotherapy cycles with LVP, followed by radiotherapy and subsequent consolidation with additional cycles of the same chemotherapy regimen. The classical protocol includes two to three cycles of LVP before radiotherapy, radiotherapy at total doses of 50–56 Gy delivered in daily fractions, and two to three additional LVP cycles as consolidation. In Spain, native L-ASP was historically used and imported as a foreign drug. From 2017, PEG-ASP became the preferred formulation due to improved availability and endorsement by cooperative groups such as PETHEMA (Programa Español de Tratamientos en Hematología). Later, in 2023, CSP (Erwinase®) was also introduced to the Spanish market. However, following the discontinuation of L-ASP and the substantially higher cost of CSP, approximately four times that of PEG-ASP, the LVP regimen was adapted to use PEG-ASP at equivalent dosing (PVP).

The chemotherapy regimen consisted of 2500 IU/m^2^ PEG-ASP administered intravenously on day 1, 1.4 mg/m^2^ vincristine administered intravenously on day 1, and 100 mg prednisone administered orally on days 1–5, repeated every three weeks. The first PVP cycle was well tolerated, leading to rapid EBV clearance. Radiotherapy was initiated after the first cycle, with 56 Gy administered in 2 Gy daily fractions over 28 sessions, with an integrated boost. The interval between completion of radiotherapy and initiation of the subsequent chemotherapy cycle was extended to eight weeks due to poor tolerance to radiotherapy, which required nasogastric tube placement and enteral nutritional support.

During the second PVP cycle, approximately five minutes after PEG-ASP administration began, she developed palmar erythema, generalized pruritus, marked malaise, sneezing, vomiting, and a presyncopal episode. The infusion was promptly discontinued, and symptoms resolved rapidly following antihistamine and hydrocortisone treatment. She remained afebrile, mildly hypotensive, and eupneic. Due to suspicion of an HSR, additional evaluations were performed by the allergology unit.

- **Skin Tests for PEG-asparaginase**:
- The intradermal test (IDT1, 7.5 mg/mL) produced a wheal increase from 3 to 6 mm with 10 mm of erythema and localized pruritus, whereas both skin prick tests (SPT1, 75 mg/mL; SPT2, 750 mg/mL) yielded negative results.
- **Latency Time (from reaction to skin test)**: 13 days.
- **Reaction Biomarkers**: Tryptase levels were not drawn at the time of the acute event.
- **Baseline Tryptase**: A baseline tryptase level, drawn during the skin test appointment, was normal.

Based on the ancillary test and the clinical presentation, an immediate (Grade 2, Brown/Grade III, RCUH) hypersensitivity reaction during the infusion of PEG-asparaginase was considered. The skin tests yielded a questionably positive result, but the clinical presentation was consistent with a Type I, IgE-mediated hypersensitivity reaction. However, other immunological mechanisms could not be excluded. The lack of acute-phase biomarker levels (e.g., tryptase) at the time of the event limits etiological confirmation and renders a definitive diagnosis uncertain.

A monitored re-exposure to PEG-asparaginase was attempted. Infusion was stopped after administration of a total dose of 125.42 IU as the patient developed general malaise, epigastric pain, and nausea during the infusion.

Vital signs were initially normal, though she experienced persistent malaise and tachycardia (heart rate 97 bpm). Rapid deterioration prompted treatment with IM epinephrine (0.5 mg), IV dexamethasone (12 mg), and IV dexchlorpheniramine (5 mg). Thirty minutes later, vital signs stabilized, though headache, chest tightness (6/10), and abdominal pain persisted. Intravenous pantoprazole (40 mg) and fluids were administered, leading to gradual recovery within 45 minutes.

The reaction did not meet the criteria for an IgE-mediated reaction, and its lack of improvement with anti-mediator further supported this interpretation. Nevertheless, both events required immediate discontinuation of treatment.

The patient was then switched to CSP, which was well tolerated. The patient remains in complete remission one year after therapy.

## 3. Retrospective Institutional Review

We retrospectively reviewed data from adult patients (age > 18 years) diagnosed with NK/T-cell lymphoma and treated with ASP-containing chemotherapy regimens between 2015 and 2025 at our institution to determine the frequency of HSR.

This revision was conducted by the ethical principles outlined in the Declaration of Helsinki and informed consent was obtained from all participants prior to inclusion. We identified six cases of NKTCL treated with ASP-based chemotherapies from 2015 to 2025. Four patients were of European origin, and the remaining two were of African descent. All patients were treated at the same institution. The median age of the cohort was 52 years (range 42–62), and 67% were male. A total of 94 asparaginase doses were administered (86 native L-ASP, 8 PEG-ASP), with only one documented clinical HSR (1/6, 16.7%).

The treatment regimens administered included SMILE, LVP, P-GemOx, and PVP. A total of 86 intravenous doses of native L-asparaginase were given across these six patients: 6000 IU/m^2^ on days 1–5 per cycle in the LVP regimen, and on days 8, 10, 12, 14, 16, 18, and 20 in the SMILE regimen. Eight doses of PEG-asparaginase were administered, five intravenously as part of the PVP regimen and three intramuscularly within the P-GemOx protocol. The patient described in our case was the first to receive CSP at our center.

Patients’ characteristics are summarized in Table 1. Of the patients treated with ASP-based regimens at our center, only one experienced this reaction.

## 4. Discussion

Although NK/T-cell lymphoma is a rare hematologic malignancy, its low incidence underscores the need for continued research to better characterize its biological behavior and expand the current understanding of the disease. Such efforts are essential to identify novel pharmacological targets, optimize therapeutic strategies, and develop reliable biomarkers.

Skin testing for L-asparaginase hypersensitivity lacks standardization, as validated diagnostic concentrations have not been established. These tests should therefore be regarded as experimental or complementary rather than definitive and highlight the urgent need for standardization. The same limitation applies to baseline tryptase measurement, which lacks diagnostic value in the absence of corresponding acute-phase levels. In our patient, both assessments were performed for exploratory purposes and were not used as diagnostic criteria.

Hypersensitivity to native and PEG-ASP is influenced by several factors. Thus, studies have shown that adults experience HSRs less frequently than children, and genetic predisposition may contribute to interindividual variability. The use of antihistamines and corticosteroids as premedication has been associated with a reduced incidence of HSRs, and reactions appear less common when ASP is administered as part of intensive chemotherapy regimens with minimal treatment interruptions. In addition, intravenous administration has been linked to a higher rate of anaphylactic reactions compared with intramuscular delivery.

However, managing asparaginase-related immune events is complicated by the cross-reactivity between native L-ASP and PEG-ASP, both *E. coli*-derived formulations that can induce HSRs [11]. Nevertheless, studies have reported that antibodies against native L-ASP are the most frequently detected (39–61%), while anti-PEG-ASP and anti- CSP antibodies are observed in a less extent (21–29% and 8–38%, respectively) [12]. Moreover, individuals producing IgG or IgM anti-ASP antibodies, rather than IgE, are less likely to develop overt hypersensitivity reactions [13].

Although limited by the sample size, the proportion of patients who developed HSR was very similar to that reported in previous studies. Specifically, in patients diagnosed with ALL or ENKTCL receiving L-ASP, hypersensitivity occurred in 12.5% of cases [14], a rate comparable to that observed in adult patients who received at least one dose of PEG-ASP in the context of ALL treatment [15].

In pediatric acute lymphoblastic leukemia (ALL), the development of antibodies against L-ASP have demonstrated to carry prognostic significance. The presence of specific IgG4 antibodies can predict L-ASP hypersensitivity, while certain chemotherapy regimens, particularly those containing cyclophosphamide, appear to reduce antibody formation, thereby allowing repeated administration of L-ASP without anaphylaxis and preventing the emergence of neutralizing antibodies [16,17,18]. Pharmacokinetic studies show that L-ASP has a plasma half-life of 18.3 ± 2.8 h (IV) and 41.7 ± 4.3 h (IM), whereas for CSP the respective values are 7.5 and 15.6 h. PEGylated-ASP, by contrast, exhibits prolonged stability due to PEGylation, with half-lives of 5.73 ± 3.24 days (IM) and 5.29 days (IV), with no significant route-dependent difference [17,19].

Importantly, the L-glutaminase coactivity of asparaginase formulations, particularly newer preparations, warrants careful assessment. The measurement of circulating glutamine and glutamate concentrations as surrogate markers of L-glutaminase activity has been proposed as a clinically useful approach [20], which could be of particular relevance in NKTCL.

## 5. Conclusions

Similarly to ALL, HSRs remain a major challenge in the treatment of NK/T-cell lymphoma with asparaginase, compromising both safety and efficacy. It is crucial to properly distinguish between the immune-mediated toxicities associated with ASP formulations, to assess their impact on this disease, and to establish appropriate management strategies based on clinical findings, available analytical tests, and any existing reference values. Establishing standardized diagnostic and grading criteria supported by clinical and laboratory evidence is critical to achieve consistent evaluation and improve patient management.

## Figures and Tables

**Table 1:** Patients’ characteristics.

Age/Sex	Stage	ECOG	Baseline Copies EBV	Chemo Scheme	1st ASP Type	Reaction	2nd ASP Type	Final Response
60/F	II-AE	1	1266	LVP	PEG-ASA	Yes	Crisantaspase	CR
42/F	IV-AE	2	516	SMILE	PEG-ASA	No	-	PR
46/M	IV	4	273	SMILE	L-ASA	No	-	NA
59/F	I-A	1	60	Multi-agent	L-ASA	No	-	CR
62/M	IV-A	0	N/R	SMILE + CHOP	L-ASA	No	-	CR
45/M	IV-B	4	2076	PVP	PEG-ASA	No	-	PR

M, male; F, female; E, extranodal, ECOG, eastern cooperative oncology group; EBV, Epstein–Barr virus; CR, complete response; PR, partial response; NA, non-available.

## Data Availability

The original contributions presented in this study are included in the article. Further inquiries can be directed to the corresponding author.

## Abbreviations

The following abbreviations are used in this manuscript:

**Abbreviation**	**Full Term/Description**
ALL	acute lymphoblastic leukemia
ASP	asparaginase
CSP	crisantaspase
ENKTCL	extranodal NK/T-cell lymphoma
HSR	hypersensitivity reactions
IDT	intradermal test
IgE	Immunoglobulin E
IgG	Immunoglobulin G
IgG4	Immunoglobulin G4
IgM	Immunoglobulin M
IM	Intramuscular
IU	International Units
IV	Intravenous
L-ASP	L-Asparaginase
LVP	L-ASP, vincristine, prednisone
NHL	non-Hodgkin lymphomas
NKTCL	NK/T-cell lymphomas
OS	overall survival
PEG-ASP	PEGylated-Asparaginase
PFS	Progression-free survival
P-gp	P-glycoprotein
PINK-B	A prognostic index for NKTCL that incorporates β-2-microglobulin.
RCUH	A grading system for hypersensitivity reactions; the term is used but not defined in the text.
SPT	skin prick test
